# *Rosmarinus Officinalis* Leaves as a Natural Source of Bioactive Compounds

**DOI:** 10.3390/ijms151120585

**Published:** 2014-11-10

**Authors:** Isabel Borrás-Linares, Zorica Stojanović, Rosa Quirantes-Piné, David Arráez-Román, Jaroslava Švarc-Gajić, Alberto Fernández-Gutiérrez, Antonio Segura-Carretero

**Affiliations:** 1 Department of Analytical Chemistry, University of Granada, c/Fuentenueva s/n, 18071 Granada, Spain; E-Mails: iborras@ugr.es (I.B.-L.); rquirantes@ugr.es (R.Q.-P.); albertof@ugr.es (A.F.-G.); ansegura@ugr.es (A.S.-C.); 2 Research and Development of Functional Food Centre (CIDAF), PTS Granada, Avda. Del Conocimiento s/n., Edificio BioRegion, 18016 Granada, Spain; 3 Faculty of Technology, University of Novi Sad, Bulevar Cara Lazara 1, 21000 Novi Sad, Serbia; E-Mails: zokastojanovic@gmail.com (Z.S.); jaroslava@tf.uns.ac.rs (J.S.-G.)

**Keywords:** *Rosmarinus officinalis*, phenolic compounds, HPLC, QTOF-MS

## Abstract

In an extensive search for bioactive compounds from plant sources, the composition of different extracts of rosemary leaves collected from different geographical zones of Serbia was studied. The qualitative and quantitative characterization of 20 rosemary (*Rosmarinus officinalis*) samples, obtained by microwave-assisted extraction (MAE), was determined by high performance liquid chromatography coupled to electrospray quadrupole-time of flight mass spectrometry (HPLC–ESI-QTOF-MS). The high mass accuracy and true isotopic pattern in both MS and MS/MS spectra provided by the QTOF-MS analyzer enabled the characterization of a wide range of phenolic compounds in the extracts, including flavonoids, phenolic diterpenes and abietan-type triterpenoids, among others. According to the data compiled, rosemary samples from Sokobanja presented the highest levels in flavonoids and other compounds such as carnosol, rosmaridiphenol, rosmadial, rosmarinic acid, and carnosic acid. On the other hand, higher contents in triterpenes were found in the extracts of rosemary from Gložan (Vojvodina).

## 1. Introduction

Rosemary (*Rosmarinus officinalis*, Lamiaceae) is a shrubby herb that grows wild in the Mediterranean basin. Today, this plant is cultivated worldwide due to its diverse uses as a common household culinary spice for flavoring. Furthermore, rosemary extracts have been widely used as a preservative in the food industry due to their inherent high antioxidant activity. In addition, it has been used as a medicinal herb for centuries, due to significant activities against many illnesses. In this sense, many major biological properties have been attributed to this plant, mainly hepatoprotective [1], antimicrobial [2,3], antithrombotic [4], diuretic [5], antidiabetic [6], anti-inflammatory [7], antioxidant [8], and anticancer [9,10,11,12]. Accordingly, it has been previously reported that rosemary extracts and their isolated components show inhibitory effects on the growth of breast, liver, prostate, lung, and leukemia cancer cells [13,14].

These potent biological activities have been attributed to the presence of many bioactive compounds in its composition. The major families found in rosemary are phenolic diterpenes including: carnosic acid, carnosol or rosmanol; flavonoids such as genkwanin, cirsimaritin or homoplantaginin; and triterpenes such as ursolic acid [15,16,17].

A type of compound present in this matrix that is currently receiving much attention are phenolic diterpenes due to a variety of health-promoting properties, such as antimicrobial [18], anti-inflammatory [19], neuroprotective [20], anti-oxidant [21], and anticancer properties [14]. In particular, carnosic acid and carnosol are two of the main antioxidant compounds present in this herb, which have been reported to have broad anticancer properties in several cell-line models, including prostate, breast, leukemia and others [12,13,22].

Another group of promising secondary plant metabolites found in rosemary is triterpenes, which present marked bioactivity, especially to treat cancer by several modes of action, among other activities. In the last decade, many studies have shown other effects that justify this expectation. In this sense, compounds such as oleanolic, betulinic, and ursolic acids, which are pentacyclic triterpenoids with anticancer, antihyperlipemic, hepatoprotective, and anti-inflammatory properties, should be highlighted [23,24].

Bioactive compounds such as flavonoids, phenolic diterpenes and triterpenes from plant sources have been traditionally extracted by a conventional solid-liquid extraction (SLE). Nevertheless, this extraction technique presents several disadvantages, mainly that it is an arduous time-consuming process, requires a high consumption of solvents, and in some cases provides low recovery. For that reason, in recent years new promising extraction methods are arising, which introduce some form of additional energy in order to facilitate the transfer of solutes from the sample to solvent in a faster process [25]. In that sense, microwave-assisted extraction (MAE) represents an alternative to conventional SLE, while improving the speed and efficiency of the extraction process and reducing the consumption of solvents [26]. MAE has been successfully used for the extraction of phenolic compounds from various plant materials, and in the case of rosemary microwaves have also been used for obtaining essential oil in steam distillation [27,28,29,30,31].

Therefore, rosemary represents an exceptionally rich source of different bioactive compounds. For this reason, the objective of this work was to study the composition of different rosemary leaves harvested in various geographical zones in Serbia in order to explore the presence of bioactive compounds. In this sense, the present study demonstrates that different extracts of this plant could be used as natural sources of several bioactive compounds, especially carnosol, carnosic acid and triterpenes, which could be useful ingredients in complementary alternative medicine and nutritional supplements, as well as natural antioxidants for food preservation.

## 2. Results and Discussion

The rosemary leaves harvested at different sites in Serbia were extracted by MAE and subsequently analyzed by high performance liquid chromatography coupled to electrospray quadrupole-time of flight mass spectrometry (HPLC–ESI-QTOF-MS). The main compounds were identified using a QTOF mass analyzer, which has proven to be a valuable detection system for characterizing phenolic compounds, since it provides mass accuracy and true isotopic pattern in both MS and MS/MS spectra. Afterwards, the compounds characterized were quantified in the extracts using commercialized standards whenever available or compounds with structure similarities.

### 2.1. Qualitative Characterization of Bioactive Compounds Present in Rosemary-Leaf Extracts

**Figure 1:**
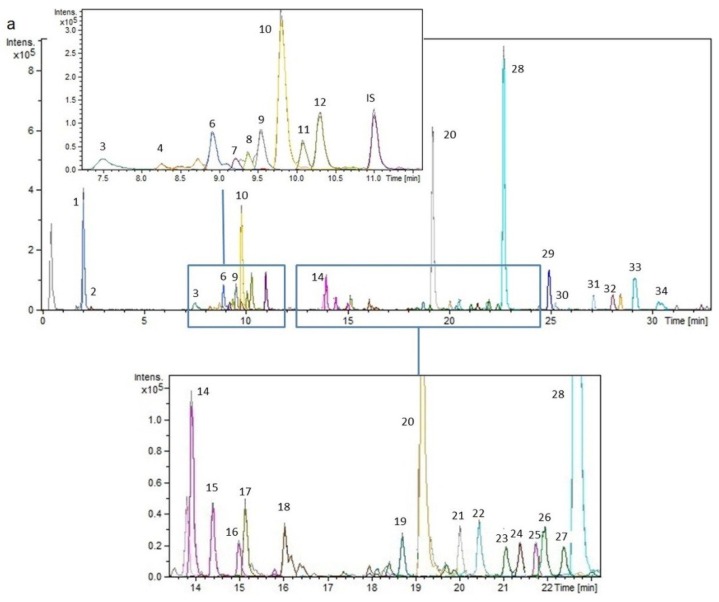

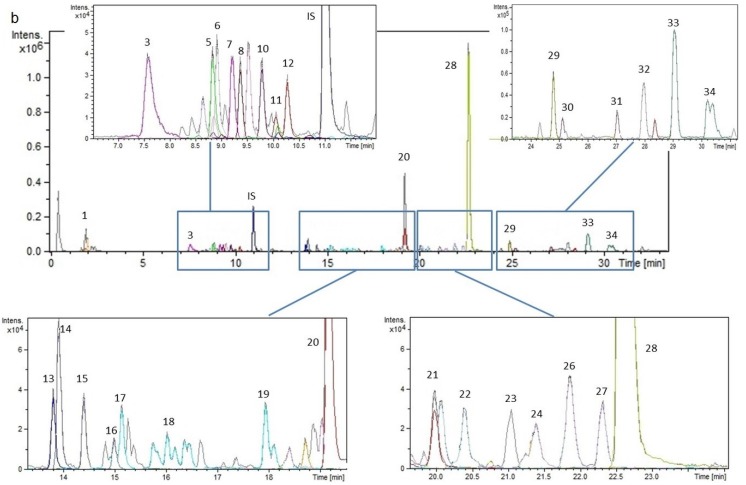
(**a**) Base Peak Chromatogram (BPC) of rosemary sample RS 2 (Sokobanja); (**b**) BPC of rosemary sample RS 10 (Gložan).

Figure 1 shows the Base Peak Chromatograms (BPC) of extracts of rosemary harvested in Sokobanja and Gložan, namely rosemary sample 2 and 10 (RS 2 and 10), respectively, as an example of the composition found in the different rosemary extracts.

The analysis of the extracts revealed the presence of 34 compounds, mainly flavonoids and phenolic diterpenes, although some organic acids and abietan-type triterpenoids were also found. The detected compounds were characterized by comparison of retention time and the MS and MS/MS spectra provided by the Q-TOF mass analyzer with those of authentic standards when available. The remaining identifications were performed by interpretation of the MS and MS/MS spectra of the detected compounds combined by the data from the literature and data bases.

Table 1 summarizes the MS data of the compounds identified, numbered according to their elution order, together with their retention times, theoretical *m*/*z*, molecular formulas, and main fragments derived from MS/MS analysis.

Most of the compounds detected in the extracts have been previously identified in rosemary leaves, such as carnosic acid, carnosol, rosmanol, its isomers epiisorosmanol and epirosmanol, as well as other derivative compounds such as methylcarnosate, epirosmanolmethylether, and 5,6,7,10-tetrahydro-7-hydroxyrosmaquinone.

Carnosic acid and carnosol were identified by comparing retention times and fragmentation patterns of those of authentic standards. Rosmanol and its isomers (*m*/*z* 345), epiisorosmanol and epirosmanol, were identified on the basis of their retention times and MS/MS spectra. In these MS/MS spectra the fragments with *m*/*z* 301 and 283 were observed, which correspond to the ions [M–H–CO_2_]^−^ and [M–H–CO_2_–H_2_O]^−^, although the first one was present only in rosmanol. These data agree with the literature concerning these compounds [32,33]. Moreover, methylcarnosate exhibited a characteristic fragmentation pattern with two ions, corresponding to the loss of CO_2_ and a subsequent loss of CH_3_ (*m*/*z* 301 and 286, respectively) [34]. Furthermore, the MS/MS spectra acquired for epirosmanol methylether was previously reported in the literature [35]. The compound 5,6,7,10-tetrahydro-7-hydroxyrosmaquinone showed ion fragments corresponding to the losses of water and the isopropyl group (*m*/*z* 283 and 358, respectively), data in agreement with other authors [36].

Other compounds typically found in rosemary, e.g. rosmarinic acid, rosmadial, rosmaridiphenol or the flavonoids homoplantaginin, cirsimaritin, genkwanin, gallocatechin, nepetrin, hesperidin, 6-hydroxyluteolin-7-glucoside, luteolin-3'-glucuronide, and two isomers of luteolin-3'-*O*-(*O*-acetyl)-β-d-glucuronide, were also detected in these extracts [15,17,32,37,38,39].

Rosmadial presented a fragmentation pattern corresponding to the losses of ethylene and propyl moieties, showing fragment ions of *m*/*z* 315 and 299, respectively [34]. For the flavonoid cirsimaritin the fragmentation pattern presented two major fragment ions at *m*/*z* 298 and 283, which are formed by two subsequent losses of methyl groups from the precursor ion [34]. The same losses were also observed for rosmaridiphenol, resulting in the fragment ion at *m*/*z* 285. Respect to gallocatechin, the fragmentation pattern presented ions at *m*/*z* 97 and 225, which are consistent with data found in literature and data bases [34]. The fragment ions found for nepetrin and 6-hydroxyluteolin-7-glucoside at *m*/*z* 315 and 301 respectively, were attributable to the loss of a glucose moiety. Similar results were found for hesperidin, which presented a major fragment ion at *m*/*z* 301 due to the loss of rutinoside [35,40]. The compound luteolin-3'-glucuronide presented a major fragment at *m*/*z* 285 from the loss of glucuronic acid [34].

Two peaks with *m*/*z* 503 were detected at retention times of 10.07 and 10.28 min corresponding to isomers of luteolin-3'-*O*-(*O*-acetyl)-β-d-glucuronide. The first isomer could be attributed to luteolin-3'-*O*-(2''-*O*-acetyl)-β-d-glucuronide due to its fragmentation pattern, which presented fragment ions at *m*/*z* 285 and 399 corresponding to [M–H–C_8_H_10_O_7_]^−^ and [M–H–C_3_H_4_O_4_]^−^. Nevertheless, for the second isomer, it was not possible to characterize their identity because the isomers luteolin-3'-*O*-(3''-*O*-acetyl)-β-d-glucuronide and luteolin-3'-*O*-(4''-*O*-acetyl)-β-d-glucuronide presented the same fragmentation pattern and both of them have been previously found in rosemary. In this case, the MS/MS analysis revealed two main fragment ions at *m*/*z* 443 and 285 corresponding to the loss of acetyl and acetyl-glucuronide moieties, respectively.

Furthermore, the analysis of the rosemary-leaf extracts showed the presence of triterpenes anemosapogenin, micromeric acid, betulinic acid and ursolic acid, which were previously described in the literature for this plant matrix [7,41,42,43].

Other compounds detected in the extract have been described in different plants belonging to the Lamiaceae family, such as quinic acid, syringic acid, rosmarinic acid-3-*O*-glucoside, [9]-shogaol and the triterpenic acids asiatic, benthamic, and augustic acids [44,45,46,47,48,49,50,51]. The MS/MS analysis of rosmarinic acid-3-*O*-glucoside showed the major fragments at *m*/*z* 477, 359 and 323 corresponding to [M–H–COO]^−^, [M–H–C_6_H_10_O_5_]^−^, and [M–H–C_9_H_10_O_5_]^−^ [52]. Lastly, two different isomers of [9]-shogaol were also characterized with the same fragmentation patterns, possessing two fragment ions for [M–H–OCH_3_]^−^ and [M–H–C_10_H_19_]^−^ (*m*/*z* 287 and 179, respectively).

**Table 1:** Compounds characterized in rosemary-leaf extracts.

Peak	Retention Time (min)	Theoretical *m/z*	Molecular Formula	Fragments	Proposed Compound
1	2.06	191.0561	C_7_H_12_O_6_	93.0338 (3.7), 127.0423 (10.2)	Quinic acid
2	2.45	197.0455	C_9_H_10_O_5_	135.0731 (100.0), 179.0516 (57.9)	Siringic acid
3	7.48	305.0666	C_15_H_14_O_7_	96.9595 (47.1), 225.1178 (100.0)	Gallocatechin
4	8.43	463.0882	C_21_H_20_O_12_	301.0414 (63.3)	6-Hydroxyluteolin-7-glucoside
5	8.85	521.1300	C_24_H_26_O_13_	323.0774 (68.7), 359.0801 (53.3), 477.1052 (100.0)	Rosmarinic acid-3-*O*-glucoside
6	8.90	477.1038	C_22_H_22_O_12_	315.0528 (36.5)	Nepetrin
7	9.20	609.1824	C_28_H_34_O_15_	301.0732 (100.0)	Hesperidin
8	9.36	461.1089	C_22_H_22_O_11_	161.0294 (32.8), 283.0258 (100.0), 297.0408 (14.3)	Homoplantaginin
9	9.53	461.0725	C_21_H_18_O_12_	285.0417 (100.0)	Luteolin-3'-glucuronide
10	9.79	359.0772	C_18_H_16_O_8_	123.0445 (19.9), 161.0244 (100.0), 179.0357 (29.6), 197.0463 (12.7)	Rosmarinic acid
11	10.07	503.0831	C_23_H_20_O_13_	285.0370 (29.1), 399.0737 (100.0)	Luteolin 3'-*O*-(*O*-acetyl)-β-d-glucuronide Isomer I
12	10.28	503.0831	C_23_H_20_O_13_	285.0418 (100.0), 443.0654 (20.0)	Luteolin 3'-*O*-(*O*-acetyl)-β-d-glucuronide Isomer II
13	13.81	313.0717	C_17_H_14_O_6_	283.0272 (100.0), 298.0503 (85.4)	Cirsimaritin
14	13.92	345.1707	C_20_H_26_O_5_	283.1718 (49.4), 301.1833 (100.0)	Rosmanol
15	14.40	345.1707	C_20_H_26_O_5_	283.1713 (48.0)	Epiisorosmanol
16	15.00	345.1707	C_20_H_26_O_5_	283.1712 (32.6)	Epirosmanol
17	15.14	283.0611	C_16_H_12_O_5_	268.0401 (100.0)	Genkwanin
18	16.04	487.3428	C_30_H_48_O_5_	–	Asiatic acid
19	18.69	359.1863	C_21_H_28_O_5_	283.1734 (35.2), 329.3651 (21.6)	Epirosmanol methyl ether
20	19.15	329.1758	C_20_H_26_O_4_	285.1885 (100.0)	Carnosol
21	20.00	329.1758	C_20_H_26_O_4_	285.1887 (100.0)	Carnosol isomer
22	20.32	343.1550	C_20_H_24_O_5_	299.1644 (12.9), 315.1634 (24.1)	Rosmadial
23	21.04	471.3479	C_30_H_48_O_4_	–	Anemosapogenin
24	21.35	315.1965	C_20_H_28_O_3_	285.1877 (42.8)	Rosmaridiphenol
25	21.83	301.1809	C_19_H_26_O_3_	258.6483 (42.6), 283.6915 (25.7)	2,3,4,4a,10,10a-Hexahidro-5,6-dihydroxy-1,1-dimethyl-7-(1-methylethyl)-9(1H)-Phenantrenone
26	21.91	471.3479	C_30_H_48_O_4_	–	Benthamic acid
27	22.35	471.3479	C_30_H_48_O_4_	–	Augustic acid
28	22.63	331.1914	C_20_H_28_O_4_	287.2078 (100.0)	Carnosic acid
29	24.84	345.2071	C_21_H_30_O_4_	286.1999 (76.1), 301.2239 (100.0)	12-metoxy-carnosic acid
30	25.14	317.2122	C_20_H_30_O_3_	179.8164 (23.8), 287.2076 (60.5)	[9]-Shogaol isomer
31	27.05	317.2122	C_20_H_30_O_3_	179.7812 (19.7), 287.2079 (54.8)	[9]-Shogaol
32	27.99	453.3347	C_30_H_46_O_3_	–	Micromeric acid
33	29.05	455.3530	C_30_H_48_O_3_	–	Betulinic acid
34	30.25	455.3530	C_30_H_48_O_3_	–	Ursolic acid

### 2.2. Quantitative Characterization of the Compounds Present in Rosemary-Leaf Extracts

Standard calibration graphs of carnosol, carnosic acid, ursolic acid, rosmarinic acid, genkwanin, luteolin-7-O-glucoside, homoplantaginin, epigallocatechin, quinic acid, syringic acid and neohesperidin were prepared using luteolin at a concentration of 5 ppm as an internal standard. The proposed method was validated with the sensitivity and precision parameters. Thus, Table 2 presents the analytical parameters: limits of detection (LODs), and quantification (LOQs), calibration range, calibration equations, and regression coefficient (*R*^2^). All the calibration curves showed good linearity for the analytes studied. LODs and LOQs for individual compounds in standard solutions were also calculated as S/N = 3 and S/N = 10, respectively, where S/N is the signal-to-noise ratio.

**Table 2:** Analytical parameters of the proposed method.

Analyte	LOD (μg/mL)	LOQ (μg/mL)	Calibration Range (μg/mL)	Calibration Equations	*R*^2^
Carnosic acid	0.018	0.06	LOQ − 70	*y* = 94.036*x* + 0.0152	0.9907
Carnosol	0.019	0.06	LOQ − 25	*y* = 84.476*x* + 0.3537	0.989
Ursolic acid	0.07	0.22	LOQ − 50	*y* = 10^6^*x* + 56483	0.9763
Rosmarinic acid	0.035	0.09	LOQ − 15	*y* = 40352*x* − 0.0142	0.9909
Genkwanin	0.014	0.04	LOQ − 15	*y* = 147.37*x* − 0.0399	0.9803
Luteolin-7-O-glucoside	0.08	0.25	LOQ − 15	*y* = 14.22*x* + 0.088	0.9818
Homoplantaginin	0.016	0.05	LOQ − 5	*y* = 62.358*x* + 0.0308	0.9912
Epigallocatechin	0.08	0.26	LOQ − 15	*y* = 12.584*x* − 0.0429	0.9887
Neohesperidin	0.03	0.1	LOQ − 15	*y* = 17.158*x* − 0.0018	0.9882
Quinic acid	0.08	0.3	LOQ − 15	*y* = 15.223*x* − 0.0244	0.9918
Syringic acid	0.24	0.8	LOQ − 15	*y* = 1.8012*x* + 0.0022	0.9909

Repeatability of the proposed method was measured as the relative standard deviation (RSD, %) in terms of concentration. Different rosemary-leaf extracts with a composition which covered all the compounds detected in the extracts were injected several times (*n* = 6) on the same day (intraday precision) and 3 times on 2 consecutive days (interday precision, *n* = 12). Intraday repeatability of the method developed for all the analytes was from 0.15% to 4.57%, whereas the interday repeatability ranged from 0.23% to 4.69%.

The compound concentrations were determined using the corrected area of each individual compound (three replicates) and by interpolation in the corresponding calibration curve. Carnosic acid, carnosol, ursolic acid, rosmarinic acid, genkwanin, homoplantaginin, quinic acid and syringic acid were quantified by the calibration curves obtained from their respective commercial standards. The remaining compounds were tentatively quantified on the basis of calibration curves from other compounds with structural similarities. The carnosic acid standard curve was used for the quantification of methylcarnosate and 5,6,7-10-tetrahydro-7-hydroxyrosmariquinone. Rosmanol, its isomers epiisorosmanol and epirosmanol, epirosmanol methylether, rosmadial, and rosmaridiphenol were quantified using the carnosol calibration curve. Ursolic acid was used to quantify asiatic, augustic, benthamic, micromeric, and betulinic acids, as well as anemosapogenin. The compounds rosmarinic acid-3-*O*-glucoside and the isomers of [9]-shogaol were expressed as rosmarinic acid. Luteolin-7-*O*-glucoside calibration curve was used to estimate the content of several compounds, in particular 6-hydroxyluteolin-7-glucoside, nepetrin, luteolin-3'-glucuronide, and the isomers luteolin-3'-*O*-(*O*-acetyl)-β-d-glucuronide. Finally, the genkwanin standard was used for cirsimaritin quantification, gallocatechin was expressed as epigallocatechin, and lastly neohesperidin was used to estimate the hesperidin content. It should be taken into account that the response of the standards can differ from that of the analytes found in the extract, and consequently the quantification of these compounds is only an estimation of their actual concentrations. Nevertheless, it can be considered a useful approximation to quantify the compounds in rosemary-leaf extracts. Table 3 summarizes the quantitative results found by HPLC–ESI-QTOF-MS for the studied extracts.

The quantitative results showed that the most abundant compounds in the rosemary-leaf extracts were phenolic diterpenes and the triterpene acids, specifically carnosic acid, carnosol, micromeric acid, betulinic acid, and ursolic acid. Moreover, quinic and syringic acids were found in high quantities in some extracts, as well as some flavonoids, such as nepetrin and gallocatechin.

As mentioned above, the presence of quinic and syringic acids were found in only some extracts, although, in the extracts where they were detected, these compounds were found at high concentrations. In particular, quinic acid was detected in the extracts RS 1, 2, 3, 4, 10, 16, 18 and 19, and syringic acid in RS 1, 3 and 18.

The extracts RS 2, 3, and 4, collected in Sokobanja, showed the highest content for most of the compounds detected: flavonoids such as homoplantaginin, gallocatechin, 6-hydroxyluteolin-7-glucoside, genkwanin, cirsimaritin, luteolin-3'-glucuronide or the isomers luteolin-3'-*O*-(*O*-acetyl)-β-d-glucuronide; together with other compounds such as quinic acid, rosmarinic acid, rosmanol, asiatic acid, rosmaridiphenol, 2,3,4,4a,10,10a-hexahidro-5,6-dihydroxy-1,1-dimethyl-7-(1-methylethyl)-9(1H)-phenantrenone, carnosol, rosmadial, carnosic acid, 12-methoxycarnosic acid, and [9]-shogaol. Moreover, the RS 4 showed very high contents of micromeric acid, betulinic acid and ursolic acid. Simultaneously with those samples harvested in Sokobanja, the RS 1 collected in Kikinda showed a high concentration of syringic acid, luteolin-3'-glucuronide and rosmanol.

On the other hand, other compounds were found in the highest concentration in the extract RS 10 harvested in the province of Gložan. These compounds were nepitrin (together with the extract RS 9, harvested in Silbaš) and the triterpenes anemosapogenin, benthamic acid, augustic acid, betulinic acid, micromeric acid, and ursolic acid. The triterpene content was remarkably high in this extract compared with the rest of rosemary extracts and proved to be a very rich source of these types of compounds, which have proved to have anti-inflammatory and anticancer activities [24].

Moreover, the extract RS 12 coming from Bačko Petrovo Selo, presented high concentrations of gallocatechin, homoplantaginin, cirsimaritin, carnosol, and the highest contents of rosmarinic acid-3-*O*-glucoside, epirosmanol, and epiisorosmanol.

Additionally, rosemary harvested in Stara Planina (RS 18) showed high contents in syringic acid, homoplantaginin, rosmarinic acid, the isomers luteolin-3'-*O*-(*O*-acetyl)-β-d-glucuronide and carnosol, together with the extracts of Sokobanja samples, as described previously.

On the other hand, the maximum contents of epirosmanol methylether and hesperidin were found in extracts collected from different provinces, specifically in extract RS 14 and 20 harvested in Rumenka and Niš, respectively.

Table 3Concentrations of compounds in rosemary-leaf extracts. (**A**) Extracts RS 1–6; (**B**) Extracts RS 7–13; (**C**) Extracts RS 14–20.Value = *X* ± SD, ND: non-detected, <LQ: below the limit of quantification.

**Table:** 

(A)							
Rt (min)	Compound	RS 1	RS 2	RS 3	RS 4	RS 5	RS 6
2.06	Quinic acid	121 ± 2	128 ± 6	154 ± 8	72 ± 5	ND	ND
2.45	Siringic acid	300 ± 20	ND	250 ± 30	ND	ND	ND
7.48	Gallocatechin	9.0 ± 0.6	11.1 ± 0.5	10.6 ± 0.3	31 ± 1	8.5 ± 0.2	4.8 ± 0.4
8.43	6-Hydroxyluteolin 7-glucoside	ND	0.81 ± 0.02	0.71 ± 0.05	ND	ND	ND
8.85	Rosmarinic acid-3-*O*-glucoside	ND	ND	ND	ND	6.14 ± 0.08	7.9 ± 0.7
8.90	Nepetrin	9.9 ± 0.1	10.0 ± 0.5	10.3 ± 0.1	3.22 ± 0.08	ND	ND
9.20	Hesperidin	2.2 ± 0.1	2.6 ± 0.1	2.8 ± 0.2	3.2 ± 0.2	1.88 ± 0.05	1.9 ± 0.2
9.36	Homoplantaginin	1.28 ± 0.06	1.51 ± 0.03	1.71 ± 0.10	1.4 ± 0.1	0.57 ± 0.03	0.40 ± 0.03
9.53	Luteolin-3'-glucuronide	10.5 ± 0.3	9.2 ± 0.4	10.5 ± 0.5	6.2 ± 0.3	0.90 ± 0.02	0.68 ± 0.06
9.79	Rosmarinic acid	15.3 ± 0.5	25 ± 1	24.3 ± 0.5	6.3 ± 0.1	9.9 ± 0.6	5.6 ± 0.2
10.07	Luteolin 3'-*O*-(*O*-acetyl)-β-d-glucuronide Isomer I	4.5 ± 0.6	5.5 ± 0.2	6.8 ± 0.3	0.42 ± 0.01	ND	ND
10.28	Luteolin 3'-*O*-(*O*-acetyl)-β-d-glucuronide Isomer II	15 ± 1	17.9 ± 0.8	19.3 ± 0.6	4.8 ± 0.3	<LQ	0.3 ± 0.1
13.81	Cirsimaritin	0.47 ± 0.06	0.58 ± 0.02	0.70 ± 0.08	0.82 ± 0.09	0.32 ± 0.02	0.19 ± 0.01
13.92	Rosmanol	2.00 ± 0.04	1.49 ± 0.03	2.48 ± 0.09	1.69 ± 0.07	0.352 ± 0.006	0.173 ± 0.005
14.40	Epiisorosmanol	0.426 ± 0.010	0.95 ± 0.05	0.90 ± 0.03	1.1 ± 0.2	0.82 ± 0.05	0.19 ± 0.03
15.00	Epirosmanol	0.23 ± 0.01	0.41 ± 0.03	0.419 ± 0.002	0.6 ± 0.1	0.271 ± 0.003	<LQ
15.14	Genkwanin	0.44 ± 0.01	0.64 ± 0.03	0.75 ± 0.04	0.70 ± 0.02	0.234 ± 0.003	0.17 ± 0.02
16.04	Asiatic acid	ND	1.65 ± 0.08	3.3 ± 0.4	1.75 ± 0.05	0.907 ± 0.009	ND
18.69	Epirosmanol methyl ether	0.158 ± 0.005	0.70 ± 0.01	0.83 ± 0.07	0.62 ± 0.08	1.16 ± 0.03	0.159 ± 0.001
19.15	Carnosol	12 ± 1	22.1 ± 0.6	22 ± 1	18.8 ± 0.8	14.05 ± 0.02	5.3 ± 0.1
20.00	Carnosol isomer	0.75 ± 0.07	0.75 ± 0.10	0.80 ± 0.07	1.00 ± 0.03	0.17 ± 0.04	0.19 ± 0.01
20.32	Rosmadial	0.30 ± 0.04	0.23 ± 0.02	0.298 ± 0.006	0.32 ± 0.02	<LQ	ND
21.04	Anemosapogenin	ND	0.457 ± 0.002	1.8 ± 0.1	2.9 ± 0.6	3.5 ± 0.2	ND
21.35	Rosmaridiphenol	0.256 ± 0.001	0.62 ± 0.02	0.49 ± 0.02	0.35 ± 0.02	0.18 ± 0.01	ND
21.83	2,3,4,4a,10,10a-hexahidro-5,6-dihydroxy-1,1-dimethyl-7-(1-methylethyl)-9(1H)-Phenantrenone	0.05 ± 0.02	0.24 ± 0.01	0.53 ± 0.01	0.49 ± 0.10	0.50 ± 0.01	ND
21.91	Benthamic acid	ND	3.7 ± 0.5	5.1292 ± 0.0002	6.1 ± 0.4	6.6 ± 0.2	ND
22.35	Augustic acid	ND	1.68 ± 0.05	2.0 ± 0.4	3.6 ± 0.1	3.6 ± 0.3	ND
22.63	Carnosic acid	24 ± 2	17.2 ± 0.8	19 ± 1	25 ± 1	2.9 ± 0.2	3.2 ± 0.5
24.84	12-metoxy-carnosic acid	2.9 ± 0.1	3.8 ± 0.1	4.0 ± 0.2	3.7 ± 0.3	0.64 ± 0.03	0.084 ± 0.005
25.14	[9]-Shogaol isomer	1.03 ± 0.02	1.41 ± 0.06	1.43 ± 0.04	1.7 ± 0.1	0.68 ± 0.01	ND
27.05	[9]-Shogaol	1.87 ± 0.08	3.4 ± 0.3	2.91 ± 0.07	1.58 ± 0.10	0.45 ± 0.02	ND
27.99	Micromeric acid	1.2 ± 0.2	8 ± 1	7.7 ± 1.0	33 ± 1	7.0 ± 0.7	ND
29.05	Betulinic acid	7.8 ± 0.6	77 ± 1	26 ± 1	70 ± 2	47 ± 2	ND
30.25	Ursolic acid	1.715 ± 0.008	21.9 ± 0.1	23 ± 1	40 ± 1	8.1 ± 0.3	ND

**Table:** 

(B)								
Rt (min)	Compound	RS 7	RS 8	RS 9	RS 10	RS 11	RS 12	RS 13
2.06	Quinic acid	ND	ND	ND	12.97 ± 0.01	ND	ND	ND
2.45	Siringic acid	ND	ND	ND	ND	ND	ND	ND
7.48	Gallocatechin	5.1 ± 0.5	4.4 ± 0.4	4.0 ± 0.4	7.7 ± 0.5	10.2 ± 0.2	15.7 ± 0.2	ND
8.43	6-Hydroxyluteolin 7-glucoside	ND	ND	ND	ND	ND	ND	ND
8.85	Rosmarinic acid-3-*O*-glucoside	10.1 ± 0.6	6.7 ± 0.5	9.5 ± 0.5	0.99 ± 0.01	17.4 ± 0.1	27 ± 1	1.10 ± 0.08
8.90	Nepetrin	ND	ND	50 ± 1	57 ± 1	ND	ND	0.270 ± 0.006
9.20	Hesperidin	1.92 ± 0.07	1.35 ± 0.10	1.56 ± 0.09	2.15 ± 0.02	2.7 ± 0.2	4.2 ± 0.1	ND
9.36	Homoplantaginin	0.66 ± 0.06	0.50 ± 0.03	0.49 ± 0.03	0.71 ± 0.03	0.71 ± 0.05	1.6 ± 0.2	0.45 ± 0.03
9.53	Luteolin-3'-glucuronide	1.31 ± 0.08	1.79 ± 0.05	0.44 ± 0.05	1.39 ± 0.08	2.6 ± 0.2	5.29 ± 0.09	0.11 ± 0.03
9.79	Rosmarinic acid	5.0 ± 0.6	7.0 ± 0.6	6.3 ± 0.4	0.855 ± 0.003	12.5 ± 0.1	20.57 ± 0.04	5.4 ± 0.3
10.07	Luteolin 3'-*O*-(*O*-acetyl)-β-d-glucuronide Isomer I	ND	ND	ND	<LQ	<LQ	ND	ND
10.28	Luteolin 3'-*O*-(*O*-acetyl)-β-d-glucuronide Isomer II	1.2 ± 0.2	0.7 ± 0.1	0.28 ± 0.08	0.33 ± 0.02	3.0 ± 0.2	8.1 ± 0.8	ND
13.81	Cirsimaritin	0.24 ± 0.02	0.27 ± 0.02	0.24 ± 0.01	0.2935 ± 0.0009	0.44 ± 0.02	0.745 ± 0.009	ND
13.92	Rosmanol	0.42 ± 0.02	0.308 ± 0.008	0.37 ± 0.03	0.65 ± 0.02	0.46 ± 0.01	1.08 ± 0.03	0.110 ± 0.006
14.40	Epiisorosmanol	0.57 ± 0.02	0.83 ± 0.02	0.97 ± 0.01	0.31 ± 0.02	0.57 ± 0.02	2.41 ± 0.07	<LQ
15.00	Epirosmanol	0.20 ± 0.02	0.257 ± 0.003	0.45 ± 0.02	0.106 ± 0.006	0.195 ± 0.003	1.02 ± 0.01	ND
15.14	Genkwanin	0.210 ± 0.007	0.26 ± 0.02	0.168 ± 0.006	0.275 ± 0.002	0.38 ± 0.02	0.476 ± 0.005	ND
16.04	Asiatic acid	ND	ND	ND	1.4 ± 0.1	<LQ	ND	ND
18.69	Epirosmanol methyl ether	0.59 ± 0.02	1.128 ± 0.001	0.385 ± 0.010	0.113 ± 0.003	0.57 ± 0.04	1.00 ± 0.07	ND
19.15	Carnosol	10 ± 2	11.7 ± 0.9	4.8 ± 0.1	5.5 ± 0.3	16.0 ± 0.2	18.5 ± 0.4	1.8 ± 0.1
20.00	Carnosol isomer	ND	ND	0.13 ± 0.02	0.39 ± 0.03	0.31 ± 0.02	0.8 ± 0.2	ND
20.32	Rosmadial	ND	ND	ND	0.116 ± 0.010	ND	0.22 ± 0.02	ND
21.04	Anemosapogenin	ND	ND	ND	4.60 ± 0.06	ND	ND	ND
21.35	Rosmaridiphenol	ND	0.15 ± 0.01	ND	0.132 ± 0.001	0.20 ± 0.01	0.3572 ± 0.0009	ND
21.83	2,3,4,4a,10,10a-hexahidro-5,6-dihydroxy-1,1-dimethyl-7-(1-methylethyl)-9(1H)-Phenantrenone	ND	0.27 ± 0.03	0.27 ± 0.02	ND	0.15 ± 0.01	0.16 ± 0.05	ND
21.91	Benthamic acid	ND	1.2 ± 0.1	ND	8.3 ± 0.2	3.6 ± 0.2	2.80 ± 0.04	ND
22.35	Augustic acid	ND	0.235 ± 0.007	ND	4.8 ± 0.2	2.2 ± 0.1	0.9 ± 0.1	ND
22.63	Carnosic acid	2.6 ± 0.6	2.11 ± 0.02	3.4 ± 0.4	14 ± 1	5.8 ± 0.8	17 ± 1	1.1 ± 0.1
24.84	12-metoxy-carnosic acid	0.30 ± 0.02	0.40 ± 0.04	0.287 ± 0.007	0.62 ± 0.02	0.52 ± 0.03	1.12 ± 0.01	ND
25.14	[9]-Shogaol isomer	ND	ND	ND	0.59 ± 0.02	0.63 ± 0.03	1.21 ± 0.03	ND
27.05	[9]-Shogaol	ND	ND	ND	0.779 ± 0.007	0.66 ± 0.07	1.264 ± 0.008	ND
27.99	Micromeric acid	1.7 ± 0.3	2.5 ± 0.2	ND	16.2 ± 0.9	11 ± 1	6.8 ± 0.6	ND
29.05	Betulinic acid	6.207 ± 0.001	7.2 ± 0.4	0.93 ± 0.05	76 ± 2	51.7 ± 0.3	39 ± 2	ND
30.25	Ursolic acid	3.0 ± 0.4	4.35 ± 0.06	0.11 ± 0.04	42 ± 1	26 ± 1	18.7 ± 0.4	ND

**Table:** 

(C)								
Rt (min)	Compound	RS 14	RS 15	RS 16	RS 17	RS 18	RS 19	RS 20
2.06	Quinic acid	ND	ND	14.2 ± 0.8	ND	46 ± 3	16.8 ± 0.5	ND
2.45	Siringic acid	ND	ND	ND	ND	210 ± 10	ND	ND
7.48	Gallocatechin	3.1 ± 0.1	6.9 ± 0.5	6.5 ± 0.5	12.2 ± 0.6	6.6 ± 0.2	3.7 ± 0.1	9.6 ± 0.7
8.43	6-Hydroxyluteolin 7-glucoside	ND	ND	ND	ND	0.19 ± 0.04	ND	ND
8.85	Rosmarinic acid-3-*O*-glucoside	2.2 ± 0.1	12.4 ± 0.8	ND	16 ± 1	ND	0.90 ± 0.02	10.4 ± 0.6
8.90	Nepetrin	0.82 ± 0.08	ND	2.3 ± 0.2	ND	9.7 ± 0.3	3.7 ± 0.3	ND
9.20	Hesperidin	1.2 ± 0.1	1.93 ± 0.05	1.3 ± 0.1	2.93 ± 0.06	2.29 ± 0.08	1.01 ± 0.05	4.4 ± 0.2
9.36	Homoplantaginin	0.417 ± 0.009	0.64 ± 0.04	0.59 ± 0.05	0.95 ± 0.09	1.4 ± 0.1	0.59 ± 0.03	1.10 ± 0.07
9.53	Luteolin-3'-glucuronide	1.24 ± 0.09	1.6 ± 0.1	7.3 ± 0.8	5.5 ± 0.4	9.3 ± 0.9	3.34 ± 0.04	3.2 ± 0.3
9.79	Rosmarinic acid	1.02 ± 0.02	10.1 ± 0.5	7.5 ± 0.6	9.7 ± 0.4	23 ± 1	9.0 ± 0.6	5.6 ± 0.2
10.07	Luteolin 3'-*O*-(*O*-acetyl)-β-d-glucuronide Isomer I	ND	ND	3.2 ± 0.2	0.367 ± 0.004	5.2 ± 0.3	1.46 ± 0.05	ND
10.28	Luteolin 3'-*O*-(*O*-acetyl)-β-d-glucuronide isomer II	0.67 ± 0.01	0.74 ± 0.06	10.1 ± 0.7	8.8 ± 0.9	16.6 ± 0.3	5.8 ± 0.3	0.89 ± 0.08
13.81	Cirsimaritin	0.17 ± 0.01	0.27 ± 0.02	0.31 ± 0.01	0.41 ± 0.05	0.51 ± 0.03	0.276 ± 0.009	0.53 ± 0.02
13.92	Rosmanol	0.10 ± 0.02	0.32 ± 0.02	0.69 ± 0.04	0.421 ± 0.008	1.44 ± 0.04	0.39 ± 0.01	0.78 ± 0.02
14.40	Epiisorosmanol	0.84 ± 0.04	0.51 ± 0.05	0.34 ± 0.01	0.26 ± 0.01	0.865 ± 0.004	0.492 ± 0.003	1.5 ± 0.1
15.00	Epirosmanol	0.103 ± 0.002	0.177 ± 0.009	0.126 ± 0.003	0.094 ± 0.007	0.41 ± 0.03	0.198 ± 0.008	0.636 ± 0.009
15.14	Genkwanin	0.16 ± 0.02	ND	0.42 ± 0.03	0.34 ± 0.04	0.56 ± 0.05	0.31 ± 0.02	0.531 ± 0.009
16.04	Asiatic acid	ND	ND	ND	ND	2.4 ± 0.3	ND	ND
18.69	Epirosmanol methyl ether	3.0 ± 0.2	0.534 ± 0.009	0.34 ± 0.01	0.15 ± 0.02	0.67 ± 0.03	0.67 ± 0.04	1.10 ± 0.04
19.15	Carnosol	3.7 ± 0.5	17.977 ± 0.002	11 ± 1	9.8 ± 0.3	18.2 ± 0.5	10 ± 1	22 ± 1
20.00	Carnosol isomer	ND	0.41 ± 0.05	0.281 ± 0.004	0.45 ± 0.01	0.57 ± 0.04	ND	0.34 ± 0.03
20.32	Rosmadial	ND	0.20 ± 0.03	ND	ND	0.226 ± 0.009	ND	0.26 ± 0.01
21.04	Anemosapogenin	ND	ND	ND	ND	0.84 ± 0.01	ND	ND
21.35	Rosmaridiphenol	ND	ND	0.206 ± 0.008	0.158 ± 0.007	0.383 ± 0.007	0.19 ± 0.01	0.37 ± 0.03
21.83	2,3,4,4a,10,10a-Hexahidro-5,6-dihydroxy-1,1-dimethyl-7-(1-methylethyl)-9(1H)-phenantrenone	ND	ND	ND	ND	ND	ND	ND
21.91	Benthamic acid	ND	1.0 ± 0.1	ND	1.3 ± 0.2	3.86 ± 0.07	ND	1.37 ± 0.07
22.35	Augustic acid	ND	<LQ	ND	0.48 ± 0.04	1.10 ± 0.03	ND	<LQ
22.63	Carnosic acid	ND	8.5 ± 0.6	6.5 ± 0.6	10.3 ± 0.6	13.7 ± 0.6	1.6 ± 0.1	4.8 ± 0.1
24.84	12-Metoxy-carnosic acid	0.13 ± 0.03	0.52 ± 0.04	4.05 ± 0.08	0.73 ± 0.09	3.15 ± 0.01	1.02 ± 0.03	1.06 ± 0.03
25.14	[9]-Shogaol isomer	ND	0.76 ± 0.05	ND	0.60 ± 0.03	1.14 ± 0.03	ND	1.11 ± 0.03
27.05	[9]-Shogaol	ND	0.85 ± 0.01	0.79 ± 0.05	0.88 ± 0.03	2.53 ± 0.08	0.51 ± 0.02	1.05 ± 0.04
27.99	Micromeric acid	ND	4.47 ± 0.06	ND	15 ± 1	5.4 ± 0.7	ND	4.1 ± 0.4
29.05	Betulinic acid	ND	26 ± 1	ND	58 ± 2	40 ± 1	ND	17 ± 1
30.25	Ursolic acid	ND	5.2 ± 0.7	ND	27 ± 1	5.5 ± 0.7	ND	3.61 ± 0.05

Finally, it can be concluded that Serbian rosemary samples harvested in Sokobanja, Bačko Petrovo Selo and Stara Planina, are very rich sources of flavonoids and typical compounds of rosemary, such as carnosic acid, carnosol, rosmadial, rosmaridiphenol, and rosmarinic acid, which are compounds with many biological properties, especially antioxidant. On the other hand, the triterpenoids as anemosapogenin, benthamic acid, augustic acid or ursolic acid were more abundant in the RS 10 collected in Gložan. These triterpenes are highly valued for their potent anticancer and anti-inflammatory activities.

The quantity, composition and ratio of plants metabolites are influenced by numerous internal and external factors, such as the plant age, climate, soil type or stress conditions that may inhibit or trigger the synthesis of specific compounds. Analyzed samples of *Rosmarinus officinalis* were collected in different geographical zones of Serbia, encompassing the altitudes from 72 to 764 m. Nevertheless, due to the fact that some compounds are in high concentration in samples collected in different altitudes, it can be assumed that this factor does not seem to seriously affect the concentration of bioactive compounds. The covered geographical area was relatively small, so the climate was very similar for all the cultivars, nevertheless the soil type for samples collected in the Northern and the Southern parts of Serbia was different further contributing to specificity of plants chemical profiles. In addition, even for the same soil type, slight shift in soil pH and composition reflected biochemical pathways in plants. Taking into consideration that analyzed rosemary samples were exposed to similar climate during their grow, it can be assumed that reported relatively modest variations in the altitude, as well as different soil composition and type, may have had significant effects of plant metabolites. This aspect should be studied in depth in future research. 

## 3. Experimental Section

### 3.1. Chemicals

All chemicals were of analytical reagent grade and used as received. Methanol for the MAE extraction of rosemary leaves was supplied by Centrohem (Stara Pazova, Serbia). Formic acid and acetonitrile for analytical chromatography were purchased from Fluka, Sigma-Aldrich (Steinheim, Germany) and Fisher Scientific (Madrid, Spain), respectively. Water was purified by a Milli-Q system from Millipore (Bedford, MA, USA). Ursolic acid, rosmarinic acid, genkwanin, luteolin, luteolin-7-*O*-glucoside, epigallocatechin and neohesperidin were from Extrasynthese (Genay, France). Carnosol, carnosic acid, and syringic acid were obtained from Fluka, Sigma-Aldrich (Steinheim, Germany). Quinic acid was supplied from Acros Organics (Geel, Belgium) and homoplantaginin from Chengdu Biopurity Phytochemicals (Chengdu, China). The stock solutions containing these analytes were prepared in dimethyl sulfoxide (DMSO) and methanol (Fisher Scientific, Madrid, Spain) and stored at −80 °C until used.

### 3.2. Samples

**Figure 2:**
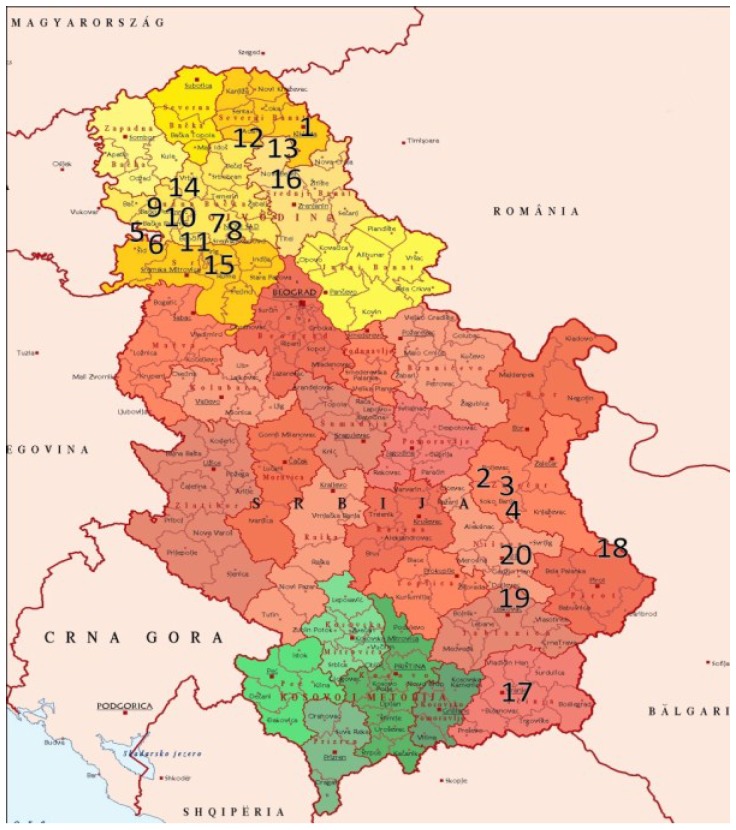
Map of Serbia where the harvesting area of each sample is marked in the corresponding geographical area.

The rosemary leaves used in this study were collected by applying non-probability haphazard sampling strategy in different geographical zones in Serbia (Figure 2), covering southern Serbia and Vojvodina and encompassing the altitudes from 72 to 764 m. The sample code, geographical origin and altitude for each sample are recovered in Table 4. From collected composite samples (~3 kg), leaves were removed and separated, participating further in the formation of representative samples. The leaves were distributed in a layer and placed in darkness at room temperature for drying, which lasted two weeks. Then, dried samples were milled and particle size was determined by sieving the ground plant material to the appropriate size (between 500 and 999 µm). The samples were stored in the freezer until used.

**Table 4:** Description of sample code, geographical area and altitude for each rosemary sample.

Sample Code	Geographical Area	Altitude
RS 1	Kikinda (Vojvodina)	73
RS 2	Sokobanja 1 (sur de Serbia)	400
RS 3	Sokobanja 2 (sur de Serbia)	415
RS 4	Sokobanja 3 (sur de Serbia)	350
RS 5	Bačka Palanka (Vojvodina)	80
RS 6	Bačka Palanka (Vojvodina)	80
RS 7	Novi Sad 1 (Vojvodina)	72
RS 8	Novi Sad 2 (Vojvodina)	80
RS 9	Silbaš (Vojvodina)	85
RS 10	Gložan (Vojvodina)	83
RS 11	Čelarevo (Vojvodina)	76
RS 12	Bačko Petrovo Selo 1 (Vojvodina)	86
RS 13	Bačko Petrovo Selo 2 (Vojvodina)	86
RS 14	Rumenka (Vojvodina)	88
RS 15	Fruška Gora (Vojvodina)	539
RS 16	Zrenjanin (Vojvodina)	80
RS 17	Vranje (sur de Serbia)	487
RS 18	Stara Planina (sur de Serbia)	764
RS 19	Leskovac (sur de Serbia)	225
RS 20	Niš (sur de Serbia)	194

### 3.3. Microwave-Assisted Extractions of Rosemary Leaves

Microwave-assisted extraction was performed by using a home-made modified domestic microwave oven (LG Electronics, Seoul, Korea). The system operated as an open-vessel multimode extraction system, allowing random dispersion of microwave radiation within the microwave cavity, so that every zone in the cavity and sample were irradiated [53]. Open cells were quartz vessels topped by a vapor condenser. The solvent was heated and refluxed through the sample and the microwaves allowed very efficient heating.

The rosemary extracts were obtained by MAE using an optimized method as described by Švarc-Gajić *et al.* [54]. In brief, the optimized procedure consisted of two pre-heating steps of 1 min in duration at 160 W and 320 W, respectively, followed by two extraction cycles with fresh solvent at 800 W for 5 min. Between all heating steps short breaks (15 s) were made in order to avoid local overheating and the risk of consistent decomposition or chemical transformation of the phenolic compounds. The solvent that provided the highest extraction yield was methanol-water 70:30 (*v*/*v*).

Died samples (2 g) were transferred to the extraction cuvettes and 25 mL of extraction solvent was added in each extraction step. The extracts obtained from two extraction cycles were joined, filtered, and evaporated to dryness using a rotary vacuum evaporator (Rotavapor R, Eph lavelle, Switzerland) and stored at −80 °C until analyzed.

### 3.4. HPLC–ESI-QTOF-MS Analysis

The rosemary-leaf extracts obtained by MAE were analyzed by HPLC–ESI-QTOF-MS. The extracts were dissolved in methanol–water 50:50 (*v*/*v*) at a concentration of 800 µg/mL. Finally, the solutions were filtered through a 0.25-μm filter and stored at −80 °C to avoid possible degradation before the HPLC analysis.

Analyses were made using a UPLC Acquity (Waters, Millford, MA, USA), equipped with a thermostat-controlled standard autosampler. The HPLC column was a Zorbax Eclipse Plus C18 (4.6 mm × 150 mm, 1.8 μm). The injection volume in the HPLC system was 5 μL and the autosampler temperature was set at 4 °C in order to avoid thermal degradation. Mobile phases A and B were water with 0.1% formic acid and acetonitrile, respectively. The separation was carried out at room temperature with a gradient elution programmed at a flow rate of 0.8 mL/min. The following multi-step linear gradient with different proportion of mobile phase B was applied: 0 min, 5% B; 12 min, 50% B; 17 min, 75% B; 22 min, 95% B; 25 min, 5% B. The initial conditions were maintained for 5 min.

The HPLC system was coupled to a microTOF-Q II mass spectrometer (Bruker Daltoniks, Bremen, Germany) via an ESI interface (Bruker Daltoniks, Bremen, Germany) operating in negative ion mode. The flow rate under chromatographic conditions was set at 0.8 mL/min. For a stable spray and consequently reproducible results, the effluent from the HPLC had to be split. In this work, a “T” type splitter was employed, and thus the flow was reduced from 0.8 to 0.2 mL/min. For all the experiments the detection was made while considering a mass range of 50–1100 *m*/*z* and using nitrogen as nebulizing and drying gas. The optimum values of the ESI–QTOF parameters were: capillary voltage, +4 kV; drying gas temperature, 210 °C; drying gas flow, 9 L/min, nebulizing gas pressure, 2 bar; funnel 1 RF, 150.0 Vpp; funnel 2 RF, 200.0 Vpp; hexapole RF, 100.0 Vpp; transfer time, 70 μs; pre-pulse storage, 7 μs. The collision-energy values for MS/MS experiments were adjusted as follows: *m*/*z* 100, 20 eV; *m*/*z* 500, 35 eV; *m/z* 1000, 50 eV.

During the execution of the HPLC method, the mass spectrometer was externally calibrated using a sodium formate cluster solution containing 10 mM sodium hydroxide and 0.1% formic acid in water:isopropanol (1:1, *v*/*v*). The mixture was injected at the beginning of each run and all the spectra were calibrated prior to compound identification. Due to the compensation of temperature drifts in the instrument, this external calibration provided accurate mass values for a complete run.

The accurate mass data of the molecular ions were processed using Data Analysis 4.0 software (Bruker Daltoniks, Bremen, Germany), which provides a list of possible elemental formulas via the Generate Molecular Formula Editor. This editor used a CHNO algorithm, which provided standard functionalities such as minimum/maximum elemental range, electron configuration, and ring-plus double-bonds equivalents, as well as a sophisticated comparison of the theoretical with the measured isotope pattern (Sigma value) for increased confidence in the suggested molecular formula.

## 4. Conclusions

In the present work, 20 rosemary plants harvested in different geographical zones of Serbia were studied in order to determine the composition of bioactive extracts. The first step was an extraction by MAE with a previously optimized procedure. Afterwards, these extracts were qualitatively and quantitatively characterized by HPLC–ESI-QTOF-MS, where the QTOF mass analyzer proved to be a valuable detection system for characterizing the phenolic compounds present in these extracts, since it provides mass accuracy and true isotopic pattern in both MS and MS/MS spectra. This coupling has enabled the tentative characterization and quantification of more than 30 different phenolic compounds, including flavonoids, phenolic diterpenes, and abietan-type triterpenes. These results highlight that extracts from Sokobanja presented the highest levels in flavonoids and other compounds such as carnosol, rosmaridiphenol, rosmadial, rosmarinic acid, and carnosic acid. On the other hand, higher contents in triterpenes were found in the extract from the rosemary collected in Gložan (Vojvodina). In conclusion, these extracts are of interest for their possible uses as ingredients in complementary alternative medicine and nutritional supplements, as well as natural antioxidants for food preservation.

## References

[1] Sotelo-Félix J.I., Martinez-Fong D., Muriel P., Santillán R.L., Castillo D., Yahuaca P. (2002). Evaluation of the effectiveness of *Rosmarinus Officinalis* (Lamiaceae) in the alleviation of carbon tetrachloride-induced acute hepatotoxicity in the rat. J. Ethnopharmacol..

[2] Del Campo J., Amiot M., Nguyen-The C. (2000). Antimicrobial effect of rosemary extracts. J. Food Prot..

[3] Bozin B., Mimica-Dukic N., Samojlik I., Jovin E. (2007). Antimicrobial and antioxidant properties of rosemary and sage (*Rosmarinus officinalis* L. and *Salvia officinalis* L., Lamiaceae) essential oils. J. Agric. Food Chem..

[4] Yamamoto J., Yamada K., Naemura A., Yamashita T., Arai R. (2005). Testing various herbs for antithrombotic effect. Nutrition.

[5] Haloui M., Louedec L., Michel J., Lyoussi B. (2000). Experimental diuretic effects of *Rosmarinus officinalis* and *Centaurium erythraea*. J. Ethnopharmacol..

[6] Bakirel T., Bakirel U., Keleş O.U., Ülgen S.G., Yardibi H. (2008). *In Vivo* Assessment of antidiabetic and antioxidant activities of rosemary (*Rosmarinus officinalis*) in alloxan-diabetic rabbits. J. Ethnopharmacol..

[7] Altinier G., Sosa S., Aquino R.P., Mencherini T., Loggia R.D., Tubaro A. (2007). Characterization of topical antiinflammatory compounds in *Rosmarinus officinalis* L.. J. Agric. Food Chem..

[8] Perez-Fons L., Garzon M.T., Micol V. (2010). Relationship between the antioxidant capacity and effect of rosemary (*Rosmarinus officinalis* L.) polyphenols on membrane phospholipid order. J. Agric. Food Chem..

[9] Lo A., Liang Y., Lin-Shiau S., Ho C., Lin J. (2002). Carnosol, an antioxidant in rosemary, suppresses inducible nitric oxide synthase through down-regulating nuclear factor-κB in mouse macrophages. Carcinogenesis.

[10] Dörrie J., Sapala K., Zunino S.J. (2001). Carnosol-induced apoptosis and downregulation of Bcl-2 in B-lineage leukemia cells. Cancer Lett..

[11] Huang S., Ho C., Lin-Shiau S., Lin J. (2005). Carnosol inhibits the invasion of B16/F10 mouse melanoma cells by suppressing metalloproteinase-9 through down-regulating nuclear factor-κB and c-Jun. Biochem. Pharmacol..

[12] Visanji J.M., Thompson D.G., Padfield P.J. (2006). Induction of G2/M phase cell cycle arrest by carnosol and carnosic acid is associated with alteration of cyclin A and cyclin B1 levels. Cancer Lett..

[13] Yesil-Celiktas O., Sevimli C., Bedir E., Vardar-Sukan F. (2010). Inhibitory effects of rosemary extracts, carnosic acid and rosmarinic acid on the growth of various human cancer cell lines. Plant Food Hum. Nutr..

[14] Johnson J.J. (2011). Carnosol: A promising anti-cancer and anti-inflammatory agent. Cancer Lett..

[15] Bai N., He K., Roller M., Lai C., Shao X., Pan M., Ho C. (2010). Flavonoids and phenolic compounds from *Rosmarinus officinalis*. J. Agric. Food Chem..

[16] Bicchi C., Binello A., Rubiolo P. (2000). Determination of phenolic diterpene antioxidants in rosemary (*Rosmarinus officinalis* L.) with different methods of extraction and analysis. Phytochem. Anal..

[17] Del Baño M.J., Lorente J., Castillo J., Benavente-García O., Marín M.P., Del Río J.A., Ortuño A., Ibarra I. (2004). Flavonoid distribution during the development of leaves, flowers, stems and roots of *Rosmarinus officinalis*. Postulation of a biosynthetic pathway. J. Agric. Food Chem..

[18] Weckesser S., Engel K., Simon-Haarhaus B., Wittmer A., Pelz K., Schempp C.M. (2007). Screening of plant extracts for antimicrobial activity against bacteria and yeasts with dermatological relevance. Phytomedicine.

[19] Poeckel D., Greiner C., Verhoff M., Rau O., Tausch L., Hörnig C., Steinhilber D., Schubert-Zsilavecz M., Werz O. (2008). Carnosic acid and carnosol potently inhibit human 5-lipoxygenase and suppress pro-inflammatory responses of stimulated human polymorphonuclear leukocytes. Biochem. Pharmacol..

[20] Kim S., Kim J., Cho H., Lee H.J., Kim S.Y., Kim S., Lee S., Chun H.S. (2006). Carnosol, a component of rosemary (*Rosmarinus officinalis* L.) protects nigral dopaminergic neuronal cells. Neuroreport.

[21] Satoh T., Izumi M., Inukai Y., Tsutsumi Y., Nakayama N., Kosaka K., Shimojo Y., Kitajima C., Itoh K., Yokoi T. (2008). Carnosic acid protects neuronal HT22 cells through activation of the antioxidant-responsive element in free carboxylic acid- and catechol hydroxyl moieties-dependent manners. Neurosci. Lett..

[22] Johnson J.J., Syed D.N., Suh Y., Heren C.R., Saleem M., Siddiqui I.A., Mukhtar H. (2010). Disruption of androgen and estrogen receptor activity in prostate cancer by a novel dietary diterpene carnosol: Implications for chemoprevention. Cancer Prev. Res..

[23] Singletary K., MacDonald C., Wallig M. (1996). Inhibition by rosemary and carnosol of 7,12-dimethylbenz[a] anthracene (DMBA)-induced rat mammary tumorigenesis and *in vivo* DMBA–DNA adduct formation. Cancer Lett..

[24] Laszczyk M.N. (2009). Pentacyclic triterpenes of the lupane, oleanane and ursane group as tools in cancer therapy. Planta Med..

[25] Garcia-Salas P., Morales-Soto A., Segura-Carretero A., Fernández-Gutiérrez A. (2010). Phenolic-compound-extraction systems for fruit and vegetable samples. Molecules.

[26] Taamalli A., Arráez-Román D., Barrajón-Catalán E., Ruiz-Torres V., Pérez-Sánchez A., Herrero M., Ibañez E., Micol V., Zarrouk M., Segura-Carretero A. (2012). Use of advanced techniques for the extraction of phenolic compounds from tunisian olive leaves: Phenolic composition and cytotoxicity against human breast cancer cells. Food Chem. Toxicol..

[27] Taamalli A., Arráez-Román D., Ibañez E., Zarrouk M., Segura-Carretero A., Fernández-Gutiérrez A. (2012). Optimization of microwave-assisted extraction for the characterization of olive leaf phenolic compounds by using HPLC–ESI-TOF-MS/IT–MS2. J. Agric. Food Chem..

[28] Proestos C., Komaitis M. (2008). Application of microwave-assisted extraction to the fast extraction of plant phenolic compounds. LWT-Food Sci. Technol..

[29] Huie C.W. (2002). A review of modern sample-preparation techniques for the extraction and analysis of medicinal plants. Anal. Bioanal. Chem..

[30] Tatke P., Jaiswal Y. (2011). An overview of Microwave Assisted Extraction and its applications in herbal drug research. Res. J. Med. Plant.

[31] Okoh O.O., Sadimenko A.P., Afolayan A.J. (2010). Comparative evaluation of the antibacterial activities of the essential oils of *Rosmarinus Officinalis* L. obtained by hydrodistillation and solvent free microwave extraction methods. Food Chem..

[32] Cuvelier M., Richard H., Berset C. (1996). Antioxidative activity and phenolic composition of pilot-plant and commercial extracts of sage and rosemary. JAOCS.

[33] Kontogianni V.G., Tomic G., Nikolic I., Nerantzaki A.A., Sayyad N., Stosic-Grujicic S., Stojanovic I., Gerothanassis I.P., Tzakos A.G. (2013). Phytochemical profile of *Rosmarinus officinalis* and *Salvia officinalis* extracts and correlation to their antioxidant and anti-proliferative activity. Food Chem..

[34] Hossain M.B., Rai D.K., Brunton N.P., Martin-Diana A.B., Barry-Ryan A.C. (2010). Characterization of phenolic composition in Lamiaceae spices by LC–ESI-MS/MS. J. Agric. Food Chem..

[35] Almela L., Sánchez-Muñoz B., Fernández-López J.A., Roca M.J., Rabe V. (2006). Liquid chromatograpic-mass spectrometric analysis of phenolics and free radical scavenging activity of rosemary extract from different raw material. J. Chromatogr..

[36] Zhang Y., Smuts J.P., Dodbiba E., Rangarajan R., Lang J.C., Armstrong D.W. (2012). Degradation study of carnosic acid, carnosol, rosmarinic acid and rosemary extract (*Rosmarinus officinalis* L.) assessed using HPLC. J. Agric. Food Chem..

[37] Herrero M., Plaza M., Cifuentes A., Ibanez E. (2010). Green processes for the extraction of bioactives from rosemary: Chemical and functional characterization via ultra-performance liquid chromatography-tandem mass spectrometry and *in vitro* assays. J. Chromatogh..

[38] Doolaege E.H.A., Raes K., Smet K., Andjelkovic M., van Poucke C., de Smet S., Verhé R. (2007). Characterization of two unknown compounds in methanol extracts of rosemary oil. J. Agric. Food Chem..

[39] Okamura N., Haraguchi H., Hashimoto K., Yagi A. (1994). Flavonoids in *Rosmarinus officinalis* leaves. Phytochemistry.

[40] Gómez-Romero M., Zurek G., Schneider B., Baessmann C., Segura-Carretero A., Fernández-Gutiérrez A. (2011). Automated identification of phenolics in plant-derived foods by using library search approach. Food Chem..

[41] Machado D.G., Bettio L.E.B., Cunha M.P., Capra J.C., Dalmarco J.B., Pizzolatti M.G., Rodrigues A.L.S. (2009). Antidepressant-like effect of the extract of *Rosmarinus officinalis* in mice: Involvement of the monoaminergic system. Prog. Neuro-Psychopharmacol. Biol. Psychiatry.

[42] Mahmoud A.A., Al-Shihry S.S., Son B.W. (2005). Diterpenoid quinones from rosemary (*Rosmarinus officinalis* L.). Phytochemistry.

[43] Collins M.A., Charles H.P. (1987). Antimicrobial activity of carnosol and ursolic acid: Two anti-oxidant constituents of *Rosmarinus officinalis* L.. Food Microbiol..

[44] De Felice A., Bader A., Leone A., Sosa S., Della Loggia R., Tubaro A., de Tommasi N. (2006). New polyhydroxylated triterpenes and anti-inflammatory activity of *Salvia Hierosolymitana*. Planta Med..

[45] Babovic N., Djilas S., Jadranin M., Vajs V., Ivanovic J., Petrovic S., Zizovic I. (2010). Supercritical carbon dioxide extraction of antioxidant fractions from selected Lamiaceae herbs and their antioxidant capacity. Innov. Food Sci. Emerg..

[46] Vrchovská V., Spilková J., Valentão P., Sousa C., Andrade P.B., Seabra R.M. (2007). Antioxidative properties and phytochemical composition of *Ballota nigra* infusion. Food Chem..

[47] Fecka I., Turek S. (2008). Determination of polyphenolic compounds in commercial herbal drugs and spices from Lamiaceae: Thyme, wild thyme and sweet marjoram by chromatographic techniques. Food Chem..

[48] Zgórka G., Glowniak K. (2001). Variation of free phenolic acids in medicinal plants belonging to the Lamiaceae family. J. Pharm. Biomed. Anal..

[49] Kasimu R., Tanaka K., Tezuka Y., Gong Z., Li J., Basnet P., Namba T., Kadota S. (1998). Comparative study of seventeen salvia plants: Aldose reductase inhibitory activity of water and MeOH extracts and liquid chromatography–mass spectrometry (LC–MS) analysis of water extracts. Chem. Pharmac. Bull..

[50] Yoshida M., Fuchigami M., Nagao T., Okabe H., Matsunaga K., Takata J., Karube Y., Tsuchihashi R., Kinjo J., Mihashi K. (2005). Antiproliferative constituents from Umbelliferae plants VII. Active triterpenes and rosmarinic acid from *Centella asiatica*. Biol. Pharm. Bull..

[51] Banno N., Akihisa T., Tokuda H., Yasukawa K., Higashihara H., Ukiya M., Watanabe K., Kimura Y., Hasegawa J., Nishino H. (2004). Triterpene acids from the leaves of *Perilla frutescens* and their anti-inflammatory and antitumor-promoting effects. Biosci. Biotechnol. Biochem..

[52] Chen X., Hu L., Su X., Kong L., Ye M., Zou H. (2006). Separation and detection of compounds in honeysuckle by integration of ion-exchange chromatography fractionation with reversed-phase liquid chromatography–atmospheric pressure chemical ionization mass spectrometer and matrix-assisted laser desorption/ionization time-of-flight mass spectrometry analysis. J. Pharm. Biomed. Anal..

[53] Mandal V., Mohan Y., Hemalatha S. (2008). Microwave assisted Extraction of curcumin by sample-solvent dual heating mechanism using taguchi L9 orthogonal design. J. Pharm. Biomed. Anal..

[54] Švarc-Gajic J., Stojanovic Z., Segura Carretero A., Arráez Román D., Borrás I., Vasiljevic I. (2013). Development of a microwave-assisted extraction for the analysis of phenolic compounds from *Rosmarinus officinalis*. J. Food Eng..

